# Molecular Design, Functional Characterization and Structural Basis of a Protein Inhibitor Against the HIV-1 Pathogenicity Factor Nef

**DOI:** 10.1371/journal.pone.0020033

**Published:** 2011-05-20

**Authors:** Sebastian Breuer, Simone I. Schievink, Antje Schulte, Wulf Blankenfeldt, Oliver T. Fackler, Matthias Geyer

**Affiliations:** 1 Abteilung Physikalische Biochemie, Max-Planck-Institut für molekulare Physiologie, Dortmund, Germany; 2 Department für Infektiologie, Virologie, Universitätsklinikum Heidelberg, Heidelberg, Germany; Karolinska Institutet, Sweden

## Abstract

Increased spread of HIV-1 and rapid emergence of drug resistance warrants development of novel antiviral strategies. Nef, a critical viral pathogenicity factor that interacts with host cell factors but lacks enzymatic activity, is not targeted by current antiviral measures. Here we inhibit Nef function by simultaneously blocking several highly conserved protein interaction surfaces. This strategy, referred to as “wrapping Nef”, is based on structure-function analyses that led to the identification of four target sites: (*i*) SH3 domain interaction, (*ii*) interference with protein transport processes, (*iii*) CD4 binding and (*iv*) targeting to lipid membranes. Screening combinations of Nef-interacting domains, we developed a series of small Nef interacting proteins (NIs) composed of an SH3 domain optimized for binding to Nef, fused to a sequence motif of the CD4 cytoplasmic tail and combined with a prenylation signal for membrane association. NIs bind to Nef in the low nM affinity range, associate with Nef in human cells and specifically interfere with key biological activities of Nef. Structure determination of the Nef-inhibitor complex reveals the molecular basis for binding specificity. These results establish Nef-NI interfaces as promising leads for the development of potent Nef inhibitors.

## Introduction

Highly active antiretroviral therapy (HAART) currently employed to treat AIDS patients consists of a combination of drugs that target the HIV enzymes reverse transcriptase, protease and integrase as well as inhibitors of virus entry. Although HAART is highly effective, its high cost and limited availability in underdeveloped areas severely limit the success of current anti-HIV therapy. Moreover, the rapid emergence of drug resistance mutants and the increased worldwide spread of such treatment resistant HIV variants pose increasing problems to effective treatment of HIV-patients [1]. One of many strategies to improve this situation is the exploitation of additional drug targets that could be added to the current regiment. Ideally, such targets comprise viral factors, since interference with host cell factors often compromises physiological function or even viability of host cells [2].

The Nef protein is an accessory gene product of HIV and SIV that is dispensable for virus spread in experimental *ex vivo* cell culture systems [3]. In infected patients or monkeys however, Nef is critical for high virus replication and disease progression. In fact, defects in the *nef* gene lead to slowly progressing or even asymptomatic infections and transgenic mice expressing Nef as the only HIV-1 gene product develop AIDS-like disease [4]–[7]. Thus, Nef is an important factor for AIDS pathogenesis. Although compounds interfering with Nef's activity would thus be of obvious global benefit, Nef is currently not a target of antiviral measures. This lack of Nef targeting reflects the limited knowledge about the mechanism by which Nef promotes virus spread and accelerates disease progression in patients. Over the last years it has become clear that Nef's impact on AIDS pathogenesis results from the combined action of several independent activities [8], [9]. First, Nef acts as a factor that prevents recognition of HIV infected cells by the host immune system (immune evasion) via a reduction of the cell surface density of bioactive MHC class I and II molecules [10] and possibly by restriction target cell motility [11]. Second, Nef alters the activation state of HIV target cells to increase their permissivity to virus replication and prolongs their life span to optimize virus production [9], [12], [13]. Third, Nef augments the infectivity of HIV particles [14]. This effect is not potentiated over several rounds of replication due to efficient but Nef-insensitive cell-to-cell spread, however accounts for the slight delay in replication kinetics observed for *nef*-deficient HIV-1 [15]. To achieve this multitude of activities, Nef has evolved as versatile adaptor for protein interactions that lacks intrinsic enzymatic activity, which allows the viral protein to affect a wide range of host cell protein sorting and signal transduction processes [3]. The lack of enzymatic activity and of a well defined *ex vivo* assay system that mirrors the complexity of Nef's biological activities *in vivo*, however, hampers the development of potent inhibitors that are effective against a broad range of Nef functions. Thus, the previously described Nef-interacting small compounds bind Nef only with relatively low affinity, display high cytotoxicity and/or interfere with only a subset of Nef interactions and functions [16]–[19]. We therefore reasoned that a rational and structure-based approach may be required for the development of an effective and multifunctional Nef inhibitor.

The structure of HIV-1 Nef is characterized by its flexible loop regions that together comprise more than half of the protein sequence [20]. Nef contains an N-terminal myristoylation site followed by an amphipathic helix for the association with cellular membranes that is thought to be critical for all Nef activities. A central PxxP motif for the interaction with SH3 domains is located within a loop section that bridges the N-terminal anchor domain to the structured core domain. This motif is essential for many interactions of Nef with host cell signal transduction cascades as well as for the downmodulation of cell surface receptors such as MHC-I and CCR5 [21], [22] and overexpression of isolated SH3 domains that bind to Nef with high affinity can interfere with its activities [23]. A dileucine based endocytosis motif (ExxxLL) is exposed at the tip of an approximately 30 residue encompassing C-terminal flexible loop to mediate the interaction with protein transport complexes, an interaction that mediates cell surface removal of CD4 and enhancement of virion infectivity by Nef [24], [25]. The recognition site for the cytoplasmic tail sequence of CD4 instead is supposed to reside within a hydrophobic groove on the core domain structure of Nef [26].

The assembly of such diverse yet highly conserved interaction motifs spread out in various flexible loop sections of the target protein renders the generation of Nef specific inhibitors that interfere with more than one individual Nef function at a time a challenging task. We thus chose a strategy aimed at wrapping the surface of Nef by the combination of different, physically linked, target domains and the iterative optimization of domain composition and linker segments.

## Results

### Molecular design of HIV-1 Nef interacting proteins

**Figure 1:**
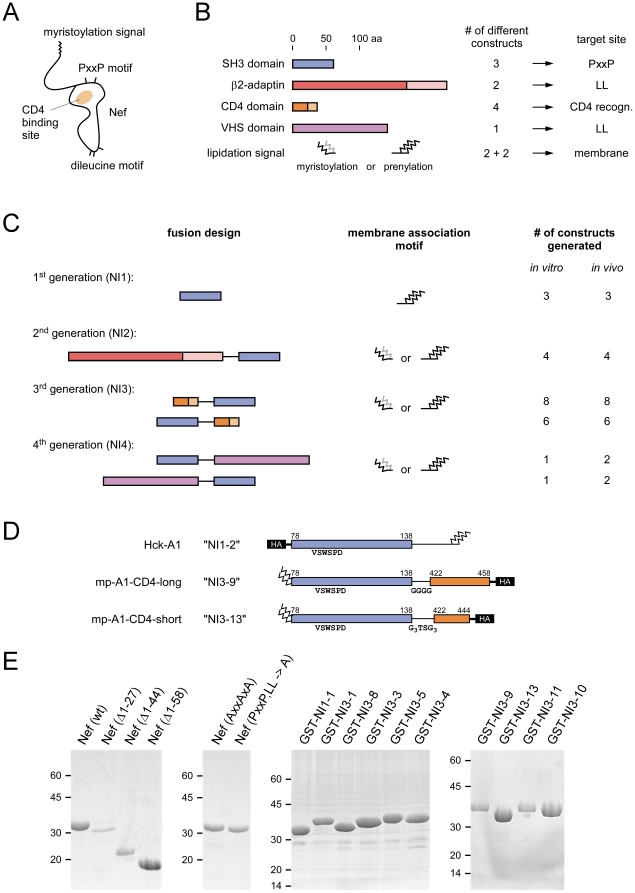
Schematic display of Nef Interacting (NI) protein design. (A) Cartoon of interaction motifs within the Nef structure. (B) Protein domains and lipidation signal sequences employed for the design of Nef interacting proteins. The number of different constructs generated and the proposed target sites are shown to the right. (C) Fusion design of four different NI generations. Different recognition domains were assembled in alternating orders and combined with various membrane association motifs to result in different expression constructs, used either for recombinant protein production or *in vivo* cell expression. (D) Display of the three high affinity binders designed to wrap the interaction surfaces of Nef. (E) SDS PAGE display of selected recombinant Nef and NI proteins used for *in vitro* binding analyses.

Nef is composed of a series of interaction motifs that spread over the surface of the protein in flexible regions at diverse sites and mediate specific interactions with host cell proteins (Figure 1A). Nef mutagenesis studies established that only simultaneous disruption of several independent protein interactions of Nef efficiently abrogates the complex array of its biological activities [8]. We therefore hypothesized that a Nef protein inhibitor has to shield several of these interaction motifs at a time. In addition, targeting multiple conserved regions simultaneously will limit the ability of resistance development. Based on the currently available knowledge about the structure-functional relationship in HIV-1 Nef, several candidate interaction motifs were considered (Figure 1B). First, we selected the SH3 domain of Hck as lead structure (referred to as ‘Nef-Interacting protein 1-1’ or ‘NI1-1’) to target the central PxxPxR motif in the poly-proline rich loop of Nef because of its relatively high affinity binding to Nef [27]. Variations of six residues in the so-called RT-loop in between the first and second β-strand of the SH3 domain further increase the affinity to Nef by 20- to 30-fold [28]. Besides human wild type Hck-SH3 (78–138) that contained the sequence E_90_AIHHE within the RT-loop, we therefore also generated the two mutant SH3 domains V_90_SWSPD and Y_90_SPFSW, termed NI1-2 and NI1-3, respectively.

Second, we included a helical region in the β-subunit of the adaptor protein complex 2 (AP-2) that was supposed to act as binding site for dileucine based sorting motifs [29]. Constructs encompassing aa 279–510 or 352–521, corresponding to either eight or six HEAT repeats of the domain structure, respectively, were generated (series NI2). Third, we used the cytoplasmic tail sequence of CD4 itself to interact with its recognition site on Nef. Two different lengths of this peptide (37 or 23 residues) were employed either as wild type sequence or in conjunction with mutation of an Lck-binding motif CQC to SQS, or mutation of the dileucine based internalization motif LL to AA (series NI3). As alternative approach to target the dileucine internalization motif in Nef, the VHS domain of the human GGA2 protein (residues 21–164) was used that was shown to bind acidic-cluster-dileucine sorting signals of the mannose-6-phosphate receptor [30], [31]. Finally, for those constructs that drive NI expression in human cells, we added a lipidation signal either as N-terminal myristoylation motif (MGxxxS) or as C-terminal farnesylation motif (CVLS) to the protein, sometimes in combination with additional palmitoylation sites, for the targeting to cellular membranes. Likewise, an HA-epitope was added always at the alternate site of lipidation in the cellular expression constructs for antibody recognition.

The selected sequences were fused in various combinations using different linker length and variable domain successions to result in four different generations of putative Nef-interacting molecules (Figure 1C). These fusion proteins were designed to interact simultaneously with multiple binding sites of Nef, leading thus to increased affinity and specificity for the viral protein. In total 23 different constructs were designed and expressed for *in vitro* studies to characterize their binding affinities to Nef and another 25 constructs for studies in human cells. An overview of the constructs generated is shown in Figure S1.

### Binding specificity between Nef and inhibitor proteins

**Figure 2:**
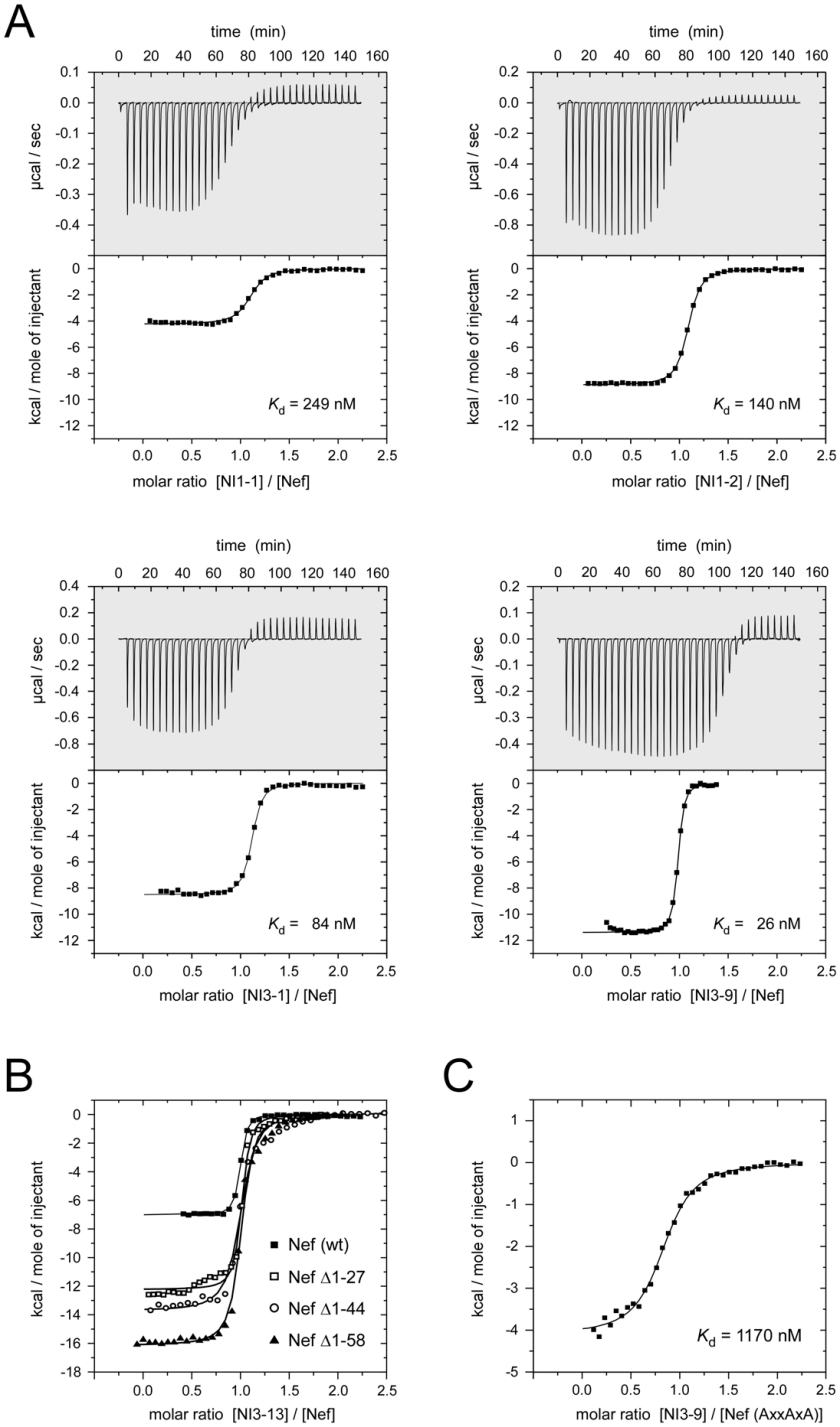
Isothermal titration calorimetric measurements between Nef and selected Nef interacting proteins. (A) Binding analyses between Nef and optimized GST-Hck-CD4 chimeras. Displayed are ITC measurements with NI1-1 (Hck, upper left), NI1-2 (Hck-RT loop optimized; upper-right), NI3-1 (CD4-Hck; lower left) and NI3-9 (Hck-CD4; lower right). (B) Contribution of the length of the N-terminal membrane anchor domain of Nef to the binding to NI3-13. (C) Binding between the prototypic Nef inhibitor NI3-9 (Hck_SH3_-polyGly-CD4) and the SH3 domain binding defective Nef (PxxPxR to AxxAxA) mutant. The dissociation constant of 1.2 µM revealed the putative contribution of the dileucine motif of CD4 to the Nef interaction. The thermodynamic parameters and the dissociation constants of the interactions are listed in Table 1.

We first purified the recombinant NI proteins (Figure 1D) and analyzed their binding capacities to HIV-1 Nef *in vitro*. Binding affinities of the direct interactions were determined by isothermal titration calorimetry (ITC), surface plasmon resonance (SPR) or fluorescence spectroscopy to compare the dissociation constants of the various fusion proteins. Nef (Δ1-44) bound to the wild type Hck-SH3 domain (termed ‘NI1-1’) with a *K*_d_ value of 1.54 µM in agreement with previous studies [32]. Mutation of the RT-loop sequence E_88_AIHHE to VSWSPD (‘NI1-2’) or YSPFSW (‘NI1-3’) led to an increase of the Nef binding affinity to 179 nM or even 48 nM, respectively, indicating the enormous contribution of this region to the PxxPxR recognition of the SH3 domain (Figure 2A and Table 1). To preserve the experimental range of interaction for further improvements we used in the following the VSWSPD sequence in the SH3 domain as a lead structure in the next generations of fusion proteins.

**Table 1:** Thermodynamic parameters of Nef-inhibitor interactions.

Interaction(cell/syringe)	T(°C)	ΔG (kcal/mol)	ΔH (kcal/mol)	TΔS (kcal/mol)	molar ratio	*K*_d_ (nM)
Nef (Δ1-44)/NI1-1	25	−7.93	−4.50±0.09	3.43	0.97	1537±195
Nef (Δ1-44)/NI1-2	25	−9.20	−8.16±0.07	1.04	0.99	179±29
Nef (Δ1-44)/NI1-3	25	−9.98	−7.99±0.07	2.00	0.95	48±11
Nef (wt)/NI1-1*	15	−8.71	−4.24±0.03	4.47	1.08	249±27
Nef (wt)/NI1-2*	15	−9.04	−8.90±0.02	0.138	1.07	140±6.7
Nef (wt)/NI3-1*	15	−9.33	−8.51±0.03	0.82	1.09	84±7.1
Nef (wt)/NI3-8*	15	−9.19	−8.20±0.04	0.99	0.93	107±10.6
Nef (wt)/NI3-5*	15	−9.49	−8.22±0.04	1.27	1.06	64±7.5
Nef (wt)/NI3-3*	15	−9.30	−8.64±0.02	0.66	1.12	88±5.4
Nef (wt)/NI3-4*	15	−8.26	−8.55±0.07	−0.288	0.97	544±59
Nef (wt)/NI3-9*	15	−10.01	−11.40±0.03	−1.39	0.97	26±2.1
Nef (wt)/NI3-13*	15	−9.74	−7.04±0.03	2.70	0.98	41±4.4
Nef (wt)/NI3-10*	15	−9.98	−11.50±0.03	−1.52	1.03	27±1.8
Nef (wt)/NI3-11*	15	−9.25	−6.91±0.02	2.35	1.10	97±5.0
Nef (AxxA)/NI3-9*	25	−8.09	−4.10±0.05	4.00	0.85	1173±111
Nef (Δ1-27)/NI3-13*	15	−9.61	−12.22±0.10	−2.61	1.00	51±9.8
Nef (Δ1-44)/NI3-13*	15	−9.26	−16.13±0.13	−6.87	1.06	96±15
Nef (Δ1-58)/NI3-13*	25	−9.31	−13.81±0.18	−4.50	0.98	149±27

*These samples were used as GST-fusion proteins.

Fusion of the proposed dileucine binding domains to the SH3 domain (generations NI2 and NI4) resulted in protein products that tended to precipitate upon purification *in vitro*. Likewise, these constructs were unstable when expressed in mammalian cells (data not shown) and could therefore not be investigated further. Attachment of the cytoplasmic domain of CD4, however, which is the smallest unit tested, turned out to be suitable for *in vitro* characterization and amenable for structure-function based binding improvements. N-terminal fusion of CD4 to SH3 by a five residue linker resulted in a dissociation constant of 84 nM of the 103 residue encompassing protein (NI3-1) and shortening of the CD4 segment to 23 residues similarly resulted in a *K*_d_ of 107 nM (NI3-8). Importantly, mutation of two cysteines (CQC to SQS) within the CD4 chain that are known to form an intermolecular zinc finger with Lck [33] did not affect the binding affinity to Nef (*K*_d_ = 84 nM and 88 nM, respectively; compare NI3-1 and NI3-3). In contrast, mutation of the dileucine motif in CD4 to alanine (NI3-4) largely diminished the binding affinity to Nef (544 nM), indicating the requirement of these residues for the interaction. The best binding affinity was indeed observed for the wild type CD4 sequence attached with a glycine-serine-rich linker of 12 residues to the SH3 domain (*K*_d_ = 64 nM; construct NI3-5, Table 1).

This observation prompted us to swap the two domains which led to the generation of constructs NI3-9 to NI3-14. Indeed, an improved dissociation constant of 26 nM was determined by ITC for the interaction of a SH3-CD4 fusion protein (NI3-9) with Nef, which is the tightest interaction described for a molecule with Nef to date. Mutation of the two cysteines caused again no effect (NI3-10), while mutation of the dileucines resulted in a four-fold decrease of affinity (NI3-11). Consistently, truncation to a fusion protein that lacks the cysteine residues, encompassing 92 residues only (NI3-13), showed still a binding affinity of 41 nM. The C-terminal residues of the CD4 segment did therefore not significantly contribute to the binary interaction. We next tested the binding of these improved proteins to a Nef mutant in which the PxxPxR motif was replaced by AxxAxA, which typically abrogates the interaction to SH3 domains. Now, the interaction with NI3-9 showed still a *K*_d_ of 1.17 µM (Figure 2C), which might be regarded as the contribution of the dileucine motif in CD4 to the binding affinity to Nef.

Finally, we tested the effect of the N-terminal flexible membrane anchor domain of Nef to the binding of the SH3-CD4 fusion proteins (Figure 2B). While for the full length Nef protein a dissociation constant to NI3-13 of 41 nM was determined, its successive truncation as Δ1-27, Δ1-44 and Δ1-58 resulted in decreased binding affinities of 51 nM, 96 nM and 149 nM, respectively. The relative change in binding affinity observed was thus largest between starting positions 28 and 45, while deletion of the N-terminal 27 residues only marginally affected the interaction. This observation is in line with the assumption that the far N-terminal region is embedded in the lipid bilayer where it may form an amphipathic helix to sustain membrane binding [34], while residues outside the core domain of Nef could still contribute to the interaction with CD4.

The tight interactions between Nef and its inhibitory proteins were confirmed by equilibrium fluorescence titration measurements using the dansyl labeled SH3-CD4 fusion variant NI3-3, that contained only one cysteine for labeling, with increasing concentrations of wild type full length Nef (Figure S2). Similarly, surface plasmon resonance was used to determine the dissociation constant between His-tagged CD4-SH3 (NI3-9) and four different concentrations of Nef that revealed a *K*_d_ of 28 nM (Figure S3). Both techniques yielded dissociation constants that were in agreement with the ITC measurements. Finally, size exclusion chromatography was performed to address whether the increase in Nef binding contributed by the CD4 segment was achieved in the same molecule in *cis* or if it would be due to complex dimerization or oligomerization in *trans*. Addition of the 11.5 kDa SH3-CD4 fusion NI3-3 to the 25 kDa Nef protein resulted indeed in a sharp elution profile at 10.5 ml, corresponding to a molecular weight of 36 kDa for the monomeric complex (Figure S4). Moreover, the content of aggregated Nef oligomers that eluted in the void volume completely vanished upon addition of the Nef inhibitor NI3-9, suggesting its tight binding and solubilization effect.

### Cellular localization of Nef interacting proteins

**Figure 3:**
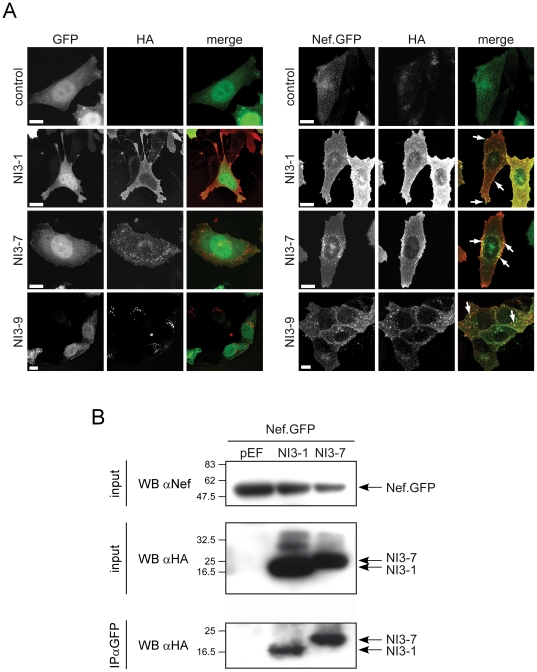
Association of Nef with NIs in human cells. (A) Localization of Nef and NIs in HeLa cells. GFP or Nef.GFP was co-expressed with an empty control vector or the indicated NIs and subjected to confocal microscopy analysis following fixation and anti-HA immunostaining. Presented are confocal sections of the middle of representative cells. Scale bar = 10 µm. Arrows indicate examples of colocalization between Nef.GFP and NIs. (B) Co-immunprecipitation of Nef.GFP and the indicated NIs from HeLa cells. Shown is a Western blot analysis of Nef.GFP and NIs in the input cell lysate (upper panel) and following anti-GFP immunoprecipitation (lower panel).

We next addressed whether these NIs also associate with the viral protein when expressed in cells. Based on the above *in vitro* analysis and their small size we focused on the 3^rd^ generation of NIs. All constructs NI3-1 to NI3-14 resulted in the expression of stable proteins to comparable levels in 293T cells (Figure S5). Expression of these NIs, either alone or in combination with Nef, did not result in apparent cytotoxicity. Confocal microscopy revealed that, when expressed alone or together with a GFP control, most NIs displayed a predominantly diffuse cytoplasmic subcellular distribution with some localization to the plasma membrane (Figure S5A). NI3-7, NI3-9 and NI3-11, however, localized prominently to intracellular membrane structures. Co-expression of Nef.GFP with the NIs resulted in marked colocalization of both proteins in most cases. This was most pronounced for constructs NI3-1, NI3-7 and NI3-9 (Figure 3A) that were subjected to further in depth analysis. Notably, the localization of Nef.GFP was altered in the presence of these constructs: while located at the plasma membrane, the cytoplasm, as well as associated with cytoplasmatic membrane vesicles when expressed alone, Nef.GFP was enriched at the plasma membrane in the presence of NI3-1 and at intracellular vesicles upon co-expression with NI3-7 and NI3-9. Most importantly and irrespective of the specific subcelluar localization, high degrees of colocalization were observed between the NIs and Nef.GFP (see arrows). Consistently, Nef.GFP was found to physically associate with NI3-1 and NI3-7 by co-immunoprecipation analysis (Figure 3B). Together these results indicate that Nef associates with the NIs in intact human cells.

### NIs interfere with biological activities of Nef

**Figure 4:**
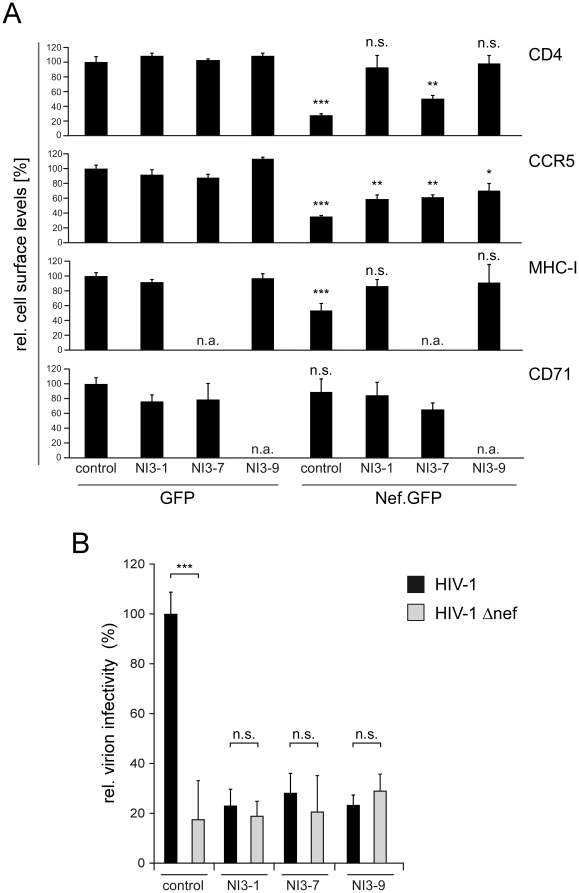
Inhibition of Nef functions by NIs in human cells. (A) FACS analysis of receptor cell surface density. Depicted are the cell surface levels of the indicated receptors on CHO) hCD4hCCR5 (for CD4 and CCR5) or HeLa-derived TZM (MHC-I, CD71) cells expressing GFP or Nef.GFP together with an empty control plasmid or the indicated NIs. Values are mean +/− STD from triplicate experiments relative to the GFP control sample that was arbitrarily set to 100%. (B) Single round of replication analysis on TZM cells for determination of virion infectivity. 2 ng p24 of HIV-1 wt or ΔNef virions produced in the presence of an empty control plasmid or the indicated NIs were used to infect TZM reporter cells. 36 h post-infection, cells were fixed, stained for β-galactosidase, and the number of blue cells was counted. Data represent mean values +/− STD from triplicate experiments plotted relative to the HIV-1 wt control that was arbitrarily set to 100% (corresponding to 200–300 blue cells/96 well). Asterisks indicate statistical significance as calculated by students *t*-test (***, p<0.0005; **,p<0.005; *, p<0.05; n.s., non significant). Statistical significance was evaluated between the corresponding GFP and Nef.GFP (A) or HIV-1 wt and ΔNef samples, respectively.

To address whether the association of NIs affects the biological properties of Nef, a series of functional assays was performed in the absence or presence of NI3-1, NI3-7 and NI3-9. The ability of Nef.GFP to reduce cell surface presentation of the receptor molecules CD4, CCR5, MHC-I and CD71 was evaluated by flow cytometry (Figure 4A). While CD71 was included as a Nef-insensitive negative control, downregulation of cell surface CD4, MHC-I and CCR5 receptors are established Nef activities that depend on select motifs in Nef: Nef-mediated downregulation of CD4 requires endocytic motifs in the C-terminal flexible loop of Nef as well as an interaction with the cytoplasmic tail of CD4 [3]. In contrast, effects of Nef on MHC-I and CCR5 are flexible loop independent but require several interaction motifs in the Nef core domain [21], [22]. Expression of the NIs alone had no significant effect on cell surface levels of CD4, CCR5, MHC-I or CD71 at these levels of expression. In contrast, Nef significantly decreased cell surface exposure of CD4, CCR5 and MHC-I but not of the negative control CD71. These effects were inhibited with variable potency by the NIs: NI3-1 and NI3-9 almost complete prevented CD4 and MHC-I downmodulation by Nef, while NI3-7 displayed only partial inhibition of Nef-mediated CD4 downregulation. All three inhibitors had intermediate effects on CCR5 downmodulation by Nef. Of note, cross-talk of inhibitors NI3-7 and NI3-9 with the fluorescent channel used for the flow cytometric analysis of MHC-I and CD71, respectively, precluded measurements of these two receptors.

Additionally, we examined the ability of the NIs to interfere with the enhancement of virion infectivity by Nef. This activity is exerted by Nef during virus particle production via a complex array of molecular determinants [8]. HIV-1 wt or Nef-deficient HIV-1 (HIV-1Δ*nef*) particles were produced in the absence or presence of NI3-1, NI3-7 or NI3-9 and the infectivity of the produced viruses was titrated on TZM indicator cells in a single round infection assay. As expected [8], [35], wt HIV-1 was approx. 5-fold more infectious than HIV-1Δ*nef* when produced in the absence of the NIs (Figure 4B). Importantly, co-expression of each of the three NIs significantly reduced the infectivity of the produced particles. No effect of the NIs was detected on the amount of particle release (data not shown) and the infectivity of HIV-1Δ*nef* was unaffected by the NIs. Thus, NI expression specifically blocked the positive effect exerted by Nef on virion infectivity during virus production. The fact that NI3-7 blocks enhancement of virion infectivity despite having little effect on CD4 downregulation induced by the viral protein indicates that, even though well correlated genetically, reduction of CD4 cell surface density may not be sufficient to explain Nef's effects on virion infectivity. Together, these results establish that NI3-1 and NI3-9 efficiently inhibit downmodulation of cell surface CD4 and MHC-I molecules as well as enhancement of virion infectivity mediated by HIV-1 Nef in human cells.

### Structure of Nef with its inhibitory proteins

**Figure 5:**
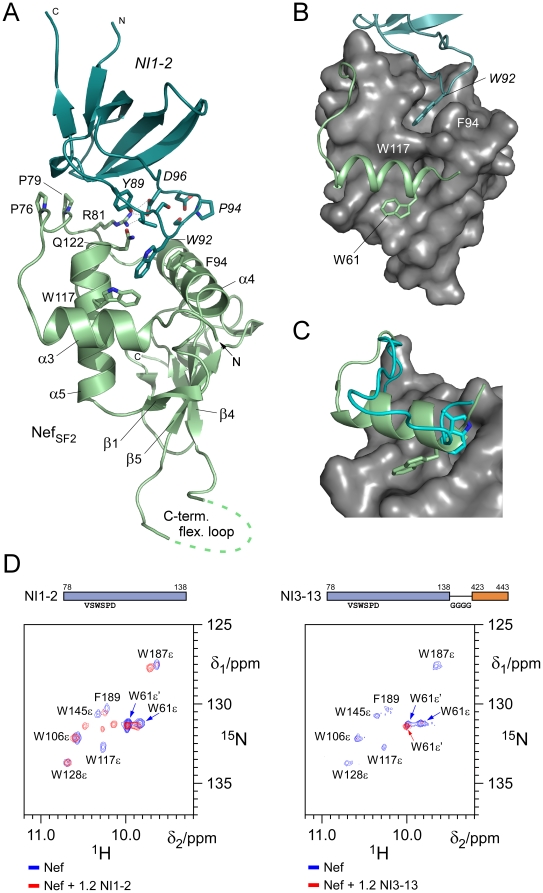
Structure of the Nef–NI1-2 inhibitor complex. (A) Ribbon display of Nef_SF2_ (44-210, Δ158-178) in complex with SH3_Hck_(VSWSPD) (NI1-2). Residues in the complex interface are highlighted. Stacking interactions between F94_Nef_ and W92_Hck_ are shown. (B) Surface display of the Nef_SF2_ core domain structure with the RT-loop sequence of Hck (top) and the newly identified helix α3 (bottom) of Nef shown as ribbon display. The gate keeper residues F94 and W117 of Nef separate two hydrophobic patches in the assembly of the α4-α5 helices that are covered by mutant W92 of Hck and W61 of Nef. (C) Superimposition of the N-terminal regions from the crystal structure determined here and the NMR structure of Nef [61]. (D) NMR mapping experiments revealed displacement of the tryptophane 61 residue by the CD4 part of NI3-13. Shown is a titration series of NI1-2 (left) or NI3-13 (right) to ^15^N-labeled Nef. Only the spectral region encompassing the HNε resonance signal of tryptophanes is shown.

To explore the structural basis of the inhibition of Nef activity by NIs we determined the crystal structures of Nef in complex with NI1-2 and NI3-13. Crystals of Nef_SF2_ (45-210, Δ158-178) bound to NI1-2 domain were refined to 2.0 Å resolution (Table 2). They contained two Nef–NI1-2 complexes in the asymmetric unit of which the first one (chains A and B) was better defined by its crystallographic electron density and is discussed in the following. The C-terminal flexible loop of Nef that contained the endocytic dileucine motif was partly truncated in the crystallized complex, nonetheless no electron density was observed starting from residues 155 to 179, in line with the assumption of a flexible loop section (Figure 5A).

**Table 2:** Crystallographic data collection and structural refinement statistics.

	Nef_SF2_ (45-210,Δ158-178) – NI1-2	Nef_SF2_ (45-210,Δ158-178) – NI3-13
**Data collection** 1		
Space group	P4_1_2_1_2	P6_1_
Cell dimensions		
*a*, *b*, *c* (Å)	65.69, 65.69, 279.07	112.46, 112.46, 130.03
α, β, γ (°)	90, 90, 90	90, 90, 120
Wavelength	0.9	0.9763
Resolution (Å)^2^	50 – 2.0 (2.1 – 2.0)	20 – 3.45 (3.55 – 3.45)
*R*_merge_	5.2 (27.5)	6.6 (30.5)
I/σ (I)	18.2 (4.4)	16.6 (3.9)
Completeness (%)	100 (100)	99.9 (100)
Redundancy	26.6 (23.4)	12.6 (12.8)
**Refinement**		
Resolution (Å)	50 – 2.0 (2.05 – 2.0)	20 – 3.45 (3.71 – 3.45)
No. reflections	40486 (3095)	12282 (2442)
*R*_work_	16.8 (20.0)	21.6 (28.0)
*R*_free_	20.5 (21.7)	24.3 (35.5)
No. atoms		
Protein	3084	2761
Water	331	–
*B*-factors		
Protein	39	132
Water	46	–
R.m.s deviations		
Bond lengths (Å)	0.026	0.016
Bond angles (°)	1.823	1.584
**PDB entry code**	3REA	3REB

1 All data sets were collected from one single crystal.

2 Values in parentheses refer to the highest resolution shell.

Interactions between the PxxP motif of Nef_SF2_ and the corresponding tryptophan-tyrosine based recognition surface on the Hck-SH3 domain of NI2-1 were mediated similarly as observed for NL4-3 Nef(T71R) to Fyn_SH3_(R96I) and LAI Nef to Fyn_SH3_ [36], [37]. In addition, two long antiparallel helices α4 and α5 (residues Y85-K98 and Q108–Q122) of Nef form a characteristic hydrophobic interface for interactions. The numbering of secondary structure elements is based on the assembly of a full length Nef structure composed of the N-terminal anchor and the C-terminal core domain [20]. The accessible surface of helices α4 and α5 is separated into two parts by the highly conserved residues F94 and W117, whose aromatic side chains oppose each other and act as gatekeepers to form a barrier in the helical region (Figure 5B). The mutant residues V_90_SWSPD of the Hck RT loop undergo tight interactions with the proximal region of these antiparallel helices, with tryptophan 92 of Hck contributing itself to the high binding affinity with 41 interactions to six different residues of Nef within a shell of 4 Å. Many of these interactions were mediated by stacking of the aromatic side chains W92_Hck_ to F94_Nef_, as well as facing interactions to W117_Nef_. In addition, R81_Nef_ in the PxxPxR motif of Nef showed direct ionic interactions to D96 of Hck and the side chain of Q122_Nef_ made tight interactions to the hydroxyl group of Y89_Hck_ (2.6 Å) and the main chain oxygen of V90_Hck_ (2.9 Å). Likewise, the hydroxyl group of S93_Hck_ was hydrogen bonded to D90_Nef_ (2.6 Å) and D95_Hck_ formed an ionic interaction to K86 of Nef (Figure S6A,B).

While the tight interactions that account for the increased affinity of mutant Hck NI1-2 compared to wild type Hck occurred all at the hydrophobic site of the two helices in Nef proximal to the PxxP motif, we now observed a previously unresolved additional helix at the N-terminus of the core domain of Nef (Figure 5B). This helix, ranging from residue A60 to E69, bound at the distal site of the two countercurrent helices with W61 of Nef interacting with hydrophobic residues L104 on the short β1 strand of Nef, I113, L114 and the methylene groups of R110 (Figure S6C). In the NMR structure of Nef_BH10_ this tryptophan residue was previously reported to exist in two different conformations with one conformation attached to its core domain structure [26] (Figure 5C). Of note, the acidic cluster motif of four successive glutamates (E66–E69), that was reported to interact with the sorting adaptor protein PACS1 [38], adopts a helical conformation on this structure element.

### The CD4 moiety interacts with the core domain structure of Nef

Crystals of Nef (45-210, Δ158-178) in complex with NI3-13 were grown under similar conditions but despite extensive testing by seeding, dehydration and additives, these crystals diffracted only to 3.5 Å resolution. Unfortunately, there was no electron density visible that would unambiguously account for the CD4 part of the inhibitory molecule. However, the resolution achieved was sufficient to show that the N-terminal chain of Nef in one complex of the asymmetric unit was bended away such that the binding site distal to the PxxP motif on face the F94–W117 gatekeeper residues could become accessible for other interactions. We therefore performed NMR spectroscopy measurements of ^15^N labeled Nef (45-210) in complex with either NI1-2 or NI3-13 as HSQC mapping experiments. The full ^15^N/^1^H HSQC overlay spectra of Nef and the Nef–NI3-13 complex are shown in Figure S7. As can be clearly seen from the tryptophan HNε region of the spectra, W61 existed in two almost equally populated conformations in the Nef protein alone but also when bound to the SH3 domain (Figure 5D, left panel, blue and red peaks). In contrast, when bound to the SH3-CD4 inhibitor construct NI3-13, W61 of Nef was fully shifted toward the low field state that was previously assigned with the open conformation of the W61 equilibrium (Figure 5D, right panel, red line) [26]. We therefore conclude that the NI3-13 molecule not only binds to the PxxPxR motif of Nef, but also interacts with the RT loop recognition site on Nef by its improved loop mutants, namely W92. In addition, NI3-13 contributes with its dileucine based sorting motif to the binding of Nef at a hydrophobic surface patch distal to the gatekeeper residues F94 and W117. Thus, NI3-13 interacts with Nef at three different but highly conserved sites leading to a substantial wrapping of the interaction surfaces of the viral protein.

## Discussion

In this study we engineered a 10.0 kDa protein (86 amino acids) that binds to HIV-1 Nef in the low nano-molar range. This protein, NI3-13, targets three highly conserved sites in Nef, namely the PxxPxR loop, the RT loop recognition site on Nef and the binding site for the CD4 dileucine based sorting signals on Nef. For tissue culture experiments the inhibitor protein was additionally targeted to lipid membranes by a myristoylation/palmitoylation motif, which is thought to further increase the binding specificity to membrane-localized Nef. Thus, a protein construct was designed that simultaneously covered three interaction sites of Nef, wrapping its functional surface sites to render the protein non-functional.

The simultaneous targeting of multiple interaction sites is a well proven approach to generate high affinity and specificity binding to a protein of interest [39], [40]. Indeed, we show here that the binding affinity of a designed protein inhibitor to HIV-1 Nef is increased from 250 to 26 nM by fusion of two structural elements and further lead optimization. This fusion protein is the tightest binder to Nef known to date and supposedly covers a large surface on the target protein. The selection of target sites within Nef was strictly dependent on high sequence conservation in HIV-1 Nef alleles of different subtypes and stages of disease. The ability to interact with the cytoplasmic tail sequence of CD4 at the plasma membrane is potentially the most conserved feature of Nef and an ancestral function in the evolution of this protein in HIV/SIV lentiviruses [11], [41]. The binding of the central PxxP motif to cellular SH3 domains adds to the diversity of Nef function in signaling and trafficking aspects. The SH3 RT loop is known to interact with a unique hydrophobic groove on Nef that significantly contributes to the specificity of the viral protein in SH3 domain recognition [27], [28]. Targeting of the inhibitor protein to cellular membranes by lipidation signals finally, further relates to the highly conserved presence of an N-terminal myristoylation signal in Nef and reduces the interaction space to a two dimensional layer.

Our functional studies in human cells provide proof-of-concept for potent inhibition of Nef function by the prototype HIV-1 Nef protein inhibitor. Importantly, this included inhibition of mechanistically independent activities such as down modulation of cell surface CD4 and MHC-I molecules, confirming the successful simultaneous interference with independent protein interactions of Nef in the absence of detectable cytotoxicity. Furthermore, inhibition of Nef was also achieved in the context of infectious HIV-1, where positive effects of Nef on the infectivity of viral progeny were blunted by the presence of the protein inhibitor. These results warrant the development of future generations of such prototype inhibitors. However, therapeutic exploitation of anti-infectives based on intracellular expression of protein inhibitors are hampered by difficulties including delivery as well as inhibitor stability and immunogenicity [42]. Similar to the recently generated inhibitors of interactions between HIV-1 integrase and its cellular cofactor LEDGF/p75 [43], such developments will build on the complex structure described here to rationally design small and cell-permeable Nef-interacting compounds. Their future functional characterization will focus on the full array of Nef activities, including analyses in primary human cells in the context of viral infection. Since one limitation of these analyses consists of the lack of potent effects of Nef on virus replication in *ex vivo* cell culture systems, ultimate validation of Nef inhibitors will require investigations in *in vivo* models such as humanized mice or HIV-1 susceptible transgenic rats [44]–[46]. Irrespective of its potential future clinical application, motif-specific intracellular Nef inhibitors at present represent valuable tools for studies of host cell interactions of the viral pathogencity factor.

The structures of Nef–NI1-2 and Nef–NI3-13 complexes described here reveal details of two conserved non-polar grooves in the HIV-1 Nef protein that are potential target sites for therapeutic intervention. Both sites are located on helices α4 and α5 of Nef that are spread apart by gatekeeper residues F94 and W117. While the site proximal to the PxxP motif harbors the central residues of the RT loop, the distal site covers a newly identified N-terminal helix of Nef (residues 60 to 69) with W61 mediating multiple interactions to hydrophobic residues L104, I113 and L114. The 8-fold increase in affinity of the Hck-SH3 RT-loop mutant VSWSPD compared to the EAIHHE wild type sequence is largely due to multiple interactions mediated by the central tryptophane residue. Importantly, this Trp residue captures the position of the mutant R96I in human Fyn kinase, which was found to be a prerequisite and key site for the interaction between the SH3_Fyn_ domain and NL4-3 Nef [27], [36]. The CD4 fragment of NI3-13 is unfortunately not resolved in the low resolution crystal structure of its complex with Nef. However, NMR spectroscopic analysis strongly support the displacement of the before mentioned newly identified N-terminal helix by the CD4 segment. Mutagenesis analyses showed that the CD4 dileucine motif is required for binding to Nef, while a C-terminal cysteine-rich motif, which was shown to form an intermolecular zinc-finger with the unique domain of Hck [33], did not contribute to the interaction. This suggested that the C-terminal residues of the CD4 segment did not contribute to the binary interaction significantly and its truncation to a fusion protein that encompassed 86 residues only (NI3-13) showed still a binding affinity of 41 nM. Together, these interaction data prove indeed the two hydrophobic cavities of Nef as potent pharmacophore target sites.

The design of protein inhibitors is often based on substrate modifications (as *e.g.* histone deacetylase inhibitors, farnesyl transferase inhibitors) or modifications of a co-enzyme/co-substrate (as *e.g.* ATP analogues for kinases) that block an enzymatic reaction. As such, inhibitors against reverse transcriptase, protease and integrase that constitute current HAART treatment target the active center of these enzymes. Combinations of these drugs are known to be highly effective, however, escape mutants that develop with time require constant monitoring and adjustment of the drug regime [2]. Other classes of inhibitors are fusion and attachment inhibitors against gp41 or CCR5 as well as maturation inhibitors targeting Gag that both impair structural factors of HIV. Fusion inhibitors have been designed in various fashions, ranging from 20 to 44 amino acid long D-peptides which interact with the tripartite coiled coil section of the glycoprotein, blocking fusion and thus entry of the virus to the target cell [47], [48]. Finally, lead inhibitory molecules that target the HIV-1 capsid and prevent assembly of infectious virus particles have been identified and are currently being developed towards therapeutic application by a design approach to achieve cell penetration [49]–[52]. While current anti-HIV infectives target enzymes or structural proteins essential to viral replication, Nef might be regarded as an accessory adaptor protein that fulfills many functions by an assembly of multiple sequence motifs. The structural and functional insight gained by the “wrapping Nef” approach described here provide valuable lead information and proof-of-concept that will guide future efforts targeted at the generation of potent and clinically applicable Nef inhibitors. Future work will match with the design, functional characterization and structure determination on Nef protein inhibitors provided in this study.

## Materials and Methods

### Cloning, expression and purification of Nef inhibitor constructs

HIV-1 Nef_SF2_ proteins (GenBank accession number K02007) were expressed in *E.coli* BL21(DE3) cells from a codon optimized plasmid with a TEV protease cleavable N-terminal His_6_-tag using pProEx HTa (Stratagene) as expression vector or pET-23d (Novagen) with a C-terminal His_6_-tag. The expression and purification of wild type Nef or N-terminally truncated Nef (Δ1-27), Nef (Δ1-44), Nef (Δ1-58), and mutant Nef (P76A, P79A, R81A) all in combination with a C-terminal cysteine to alanine mutation (C210A) was performed similarly as described [53]. Briefly, bacterial cells were cultured in LB medium containing 100 µg/ml ampicillin at 30°C and induced at OD_600_ = 0.8 with 0.5 mM IPTG for additional 5 hrs. For NMR experiments Nef (Δ1-44) has been cultured in presence of minimal medium containing 1 g/l ^15^NH_4_Cl. The harvested cells were resuspended in 20 mM Tris/HCl (pH 8.0), 500 mM NaCl, 5 mM ß-mercaptoethanol, 1 mM PMSF, lysed by fluidizer and cleared by spinning for 45 min at 30,000 g. The fusion proteins were separated from the bacterial proteins by affinity chromatography using Ni-NTA resin (Qiagen) and size exclusion chromatography on a Superdex 75 column (Amersham Bioscience), analyzed by SDS Page and MALDI-TOF mass spectrometry and stored in 10 mM Tris/HCl (pH 8.0), 50 mM NaCl at −80°C.

Fusion constructs of human Hck-SH3 domains (AC: M16591) with either the adaptor protein β2 subunit (M34175), the cytoplasmatic tail of human CD4 (M12807) or the VHS domain of GGA2 (AF233522), were generated by PCR using a cassette system of restrictions sites with *Nco*I and *Eco*R1 at the 5′ and 3′ end, respectively, and *Bam*HI or *Spe*I in between the domains. Typically, a glycine rich amino acid linker as GGGTSGGG or GGGTSGGGSGGG was cloned in between the domains. For *in vitro* experiments, proteins were expressed with an N-terminal glutathione S-transferase (GST-) tag using the pGEX-4T1 Tev vector system (Amersham Bioscience) in LB medium containing 100 µg/ml ampicillin at 30°C and induced at an OD_600 nm_ = 0.8 with 0.5 mM IPTG for additional 8 h. The harvested cells were resuspended in 20 mM Tris/HCl, pH 8.0, 500 mM NaCl, 2 mM GSH, 1 mM EDTA, 0.5 mM DTE and 1 mM PMSF, lysed by fluidizer and cleared by spinning for 45 min at 30,000 g. By affinity chromatography utilizing GSH resin (Amersham Bioscience) and size exclusion chromatography on a Superdex 75 column (Amersham Bioscience) the fusion proteins were separated from the bacterial proteins, analyzed by SDS Page and MALDI-TOF mass spectrometry and stored in 10 mM Tris/HCl, pH 8.0, 50 mM NaCl at −80°C.

For cellular expression assays, Nef interacting proteins were optionally targeted to lipid membranes by an N-terminal myristoylation signal MGSRWSK, often in combination with a palmitoylation motif (S3C), since myristoylation alone was often shown to be not sufficient for stable membrane association [34], [54]. Alternatively, a C-terminal palmitoylation and farnesylation sequence KLRKLNPPDESGPGCMSCKCVLS was added that was adopted from human H-Ras.

### Isothermal titration calorimetry

Isothermal titration calorimetry (ITC) experiments were performed in ITC buffer (10 mM Tris/HCL, pH 8.0, 100 mM NaCl) at 15 or 25°C using a Microcal VP-ITC apparatus (MicroCal, Northampton, MA), similarly as described [55]. Prior to the titration experiments, Nef and inhibitor proteins were equilibrated in ITC buffer by dialysis or resuspension and exhaustively degassed. For all reactions a solution of 400 to 500 µM inhibitor protein was injected in 25–40 steps of 4 or 8 µl volume to a solution of 40 to 50 µM Nef protein placed in the measurement cell. Each titration experiment was corrected by a control titration of the ligand into ITC buffer. Experimental data were fitted using the nonlinear least-squares algorithm with ORIGIN software (MicroCal).

### Surface plasmon resonance

Glutathione S-Transferase tagged inhibitor protein NI3-9 at 0.4 mg/ml concentration was immobilized on the surface of a SPR-based biosensor CM5 chip (BIAcore AB, Sweden) via an anti-GST antibody (BIAcore AB, Sweden) to record binding to Nef in real time. The binding experiments were performed using a SPR-based biosensor (BIAcore 1000, BIAcore AB, Sweden) in SPR buffer (10 mM HEPES pH 8.0, 150 mM NaCl, 0.005% IGEPAL) at 25°C. The association of a dilution series of hexa-histidine tagged Nef (400–1000 nM) was monitored over time at a flow rate of 5 µl/min for 380 s. Dissociation was analyzed by washing with 1 M guanidine hydrochlorid buffer for 240 min. Rate constants were calculated by using the evaluation software supplied by the manufacturer.

### Size exclusion chromatography

Analytical gel filtration of Nef and the complex of Nef and NI3-3 was performed using an HPLC (Waters) system and a Superdex S75 (10/30) column (Amersham Biosciences). 100 µl of 1 mg/ml protein solution was loaded with an elution volume of 1 ml per minute in 20 mM Tris/HCL (pH 8.0), 50 mM NaCl onto the S75 column at room temperature. The optical density was monitored at a wavelength of 280 nm over the time course of the experiment.

### Complex crystallization and structure determination

For crystallization 15 mg ml^−1^ of purified Nef–NI1-2 protein complex was stored in 30 mM Hepes buffer, pH 8.0, and 50 mM NaCl. Crystallization drops were set at a 1∶1 ratio with the reservoir solution containing 10% (w/v) PEG 8000, 0.2 M MgCl_2_, 15 mM MnCl_2_, 5% (v/v) ethylene glycol, 100 mM Tris buffer, pH 7.0, and subjected to hanging drop vapor diffusion at 12°C. Crystals appeared within three days and grew to a size of approx. 100 * 100 * 50 µm. They were briefly washed in mother liquor supplemented with 10% sucrose, 10% xylitol and 15% ethylene glycol prior to flash-cooling in liquid nitrogen. Nef–NI3-13 was crystallized in a similar fashion, using a reservoir of 0.1 M Hepes pH 7.0, 0.05 M sodium citrate, 15% isopropanol and 30% glycerol as a cryoprotectant.

Diffraction data were collected on beamline X10SA of the Swiss Light Source (SLS, Villigen, Switzerland) equipped with a MAR 225 CCD detector. The XDS package [56] was used for data reduction and the structure of the Nef–NI1-2 complex was solved by molecular replacement using the Nef-FynSH3 complex (PDB entry 1EFN [36]) as a search model in PHASER [57]. The model was refined to 2.0 Å resolution by alternating cycles of manual rebuilding in COOT [58] and minimization in REFMAC5 [59], defining each of the four chains contained in the asymmetric unit as a separate TLS body. The Nef–NI3-13 complex was solved by molecular replacement with the refined structure of Nef–NI1-2 and the structure was refined against 3.45 Å resolution data using COOT and phenix.refine [60]. Due to the low resolution, individual atomic displacement factor refinement was not performed and B-factors were only derived from TLS refinement, refining the four chains as separate TLS groups. Data collection and refinement statistics are summarized in Table 2. Molecular diagrams were drawn using PyMOL (http://pymol.sourceforge.net/).

### Nuclear magnetic resonance spectroscopy

NMR spectroscopy experiments were carried out with a Varian Inova 600 MHz spectrometer at 25°C in 5 mM Tris/HCL (pH 8.0) buffer and 8% D_2_O. The 2D ^1^H/^15^N HSQC spectra were obtained by titrating 0.4–0.6 mM Nef with either NI1-2 or the interacting fusion protein NI3-13 to a final concentration of 0.8 mM. Spectra were analyzed based on the resonance line assignments of Nef, alleles BH10 and SF2 [61]–[63] using the program AURELIA [64].

### Cells and Reagents

293T and TZM cells were purchased from ATCC (Teddington, UK). 293T, TZM (JC53BL), and TZM clone 13 cells (subcloned for high expression of MHC-I by fluorescence-activated cell sorting) were maintained in DMEM high medium (GIBCO) supplemented with 10% fetal calf serum (Invitrogen), L-glutamine and antibiotics (both from GIBCO) as described [8]. CHO hCD4hCCR5 (stably expressing hCD4 and hCCR5) [65] cells were cultured in RPMI 1640 medium supplemented accordingly.

### Mammalian expression plasmids

The expression plasmid encoding wild-type HIV-1_SF2_ Nef as a GFP fusion protein (from the pEGFP-N1 vector; Clontech) has been described previously [65]. The proviral construct expressing the *nef* gene of HIV-1 SF2 in the backbone of the HIV-1_NL4-3_ proviral clone has been described [8].

### Immunofluorescence

To visualize the subcellular localization of Nef.GFP and HA-tagged inhibitors, CHO hCD4hCCR5 cells were seeded onto glass coverslips and transfected with corresponding expression plasmids. 36 hours later, cells were fixed with 4% paraformaldehyde/phosphate-buffered saline (10 min at room temperature), permeabilized with 0.1% Tx-100, blocked for 1 h with 1% BSA in PBS, and stained with HA-antibody (F7; Santa Cruz Biotechnology) followed by appropriate fluorescent secondary antibody (Alexa568, Molecular Probes). Following extensive washing, cells were mounted with Histoprime (Linaris). Confocal microscopy images were acquired with a LSM 510 confocal laser scanning microscope (Zeiss). Final images were processed in PhotoshopCS2 (Adobe Systems).

### Western Blotting

For Western blot analysis, post-nuclear supernatants were boiled in SDS sample buffer, separated by 10% SDS-PAGE and transferred to a nitrocellulose membrane. Protein detection was performed following incubation with appropriate first and secondary antibodies using the super signal pico detection kit (Pierce) according to the manufacturer's instructions.

### Co-Immunoprecipitation

CHO hCD4hCCR5 cells were lysed in IP-lysis buffer (25 mM Tris/HCl pH 7.4; 150 mM NaCl, 1 mM EDTA, 1% NP-40) containing protease inhibitors. After removal of nuclei and cell debris by centrifugation at 1.000 g for 10 min at 4°C, lysates were incubated over night at 4°C with prepared GFP-protein A-Sepharose beads (anti-GFP-antibodies incubated with protein A-Sepharose (Pharmacia) for 2 h at room temperature in IP-buffer 1 (25 mM Tris/HCl pH 7.4; 150 mM NaCl, 1 mM EDTA, 0.5% NP-40) and washed in IP-buffer 2 (25 mM Tris/HCl pH 7.4; 150 mM NaCl, 1 mM EDTA, 0.5% NP-40, 1% BSA). Samples were pelleted, washed with IP-buffer 0 (25 mM Tris/HCl pH 7.4; 150 mM NaCl, 1 mM EDTA), and resuspended in sample-buffer. After SDS-PAGE, western blotting with anti-HA-mAb was performed.

### Flow cytometry

24 h post-transfection surface molecules of Chinese hamster ovary (CHO) hCD4hCCR5 or TZM clone 13 cells were stained, respectively as reported previously [22], [65]. Staining was performed at 4°C for 30 min in fluorescence-activated cell sorting (FACS) medium (3% FCS, 0.05% sodium azide in PBS) with anti-hCD4-APC, anti-hCCR5-APC (both BD Pharmingen), anti-CD71 (H68.4; Zymed Laboratories), and anti-MHC-I (W6/32-FITC, Sigma-Aldrich) antibodies, respectively. For unconjugated anti-CD71 mAb, secondary staining was performed with APC-conjugated goat anti-mouse antibody (Jackson ImmunoResearch Laboratories). Samples were analyzed by flow cytometry using a FACSCalibur cytometer (Becton Dickinson).

### Virus stocks and virion infectivity

Virus stocks were generated by transfection of proviral HIV plasmids into 293T cells by the Ca-phosphate method. Two days after transfection, culture supernatants were harvested. The CA concentration of concentrated stocks was determined by CA antigen enzyme-linked immunosorbent assay (ELISA) as described [66]. Relative infectivity of HIV-1 particles was determined by CA ELISA and a standardized 96-well TZM blue cell assay was performed as described earlier [65]. Briefly, infections were carried out in triplicates with 2 ng CA input virus. 36 hours post infection, cells were fixed, stained for ß-galactosidase and the number of blue cells was determined by microscopy.

### Accession codes

Atomic coordinates and structure factors of the Nef-inhibitor complexes were deposited in the Protein Data Bank with accession codes 3REA for Nef–NI1-2 and 3REB for Nef–NI3-13, respectively.

## Supporting Information

Figure S1 **Schematic display of the four generations of inhibitor constructs used in cellular experiments.** Domain boundaries, membrane targeting sites and sequence epitopes attached for antibody recognition *in vivo* are indicated. Domain boundaries for the human Hck SH3 domain are given according to the UniProt database entry P08631-1, described as isoform 1 of protein product p60-Hck. Residue numbering of human CD4 is assigned according to UniProt protein entry P01730. All protein products besides NI3-6 were targeted to cellular membranes either by an N-terminal myristoylation motif or a C-terminal farnesylation signal, partly in combination with an additional palmitoylation signal for increased membrane association, which is schematically indicated as carbon structure (zigzag lines).
(TIF)

Figure S2 **Fluorescence spectroscopy analysis of the binding interaction between Nef and NI3-3.** (A) Emission spectra of fluorescence-labeled NI3-3 Hexim1 alone and in complex with Nef (1-210) indicate complex formation by an increase and shift in fluorescence emission. (B) Analysis of an equilibrium titration series of NI3-3-EDANS with increasing concentrations of Nef (1-210) indicated a dissociation constant *K*_d_ of 37 nM. The relative fluorescence intensity was corrected for the dilution effects.
(TIF)

Figure S3 **Surface plasmon resonance measurements of Nef binding to an inhibitor of the third generation.** (A) Nef_SF2_ (45-210) was floated at four different concentrations (0.4 to 1.0 µM) over GST-NI3-9 which was immobilized on the chip surface of the SPR biosensor. Following the association step Nef protein was washed off after 400 s by Guanidine hydrochlorid. (B) The concentration dependent analysis of the association and dissociation reaction revealed a dissociation constant of 28 nM for the Nef–GST-NI3-9 interaction.
(TIF)

Figure S4 **Size exclusion chromatography of Nef and the Nef–NI3-3 protein complex.** While Nef (45-210) elutes at its expected size of ∼28 kDa as similarly observed before [53], the Nef–NI3-3 complex elutes at a molecular weight of approximately 38 kDa. These observations suggest a heterodimeric but not a heterotetrameric complex assembly, indicating that the gain in affinity by the CD4 fraction in NI3-3 is achieved by the interaction with the same Nef molecule. Note that the small portion of oligomerized Nef in the void volume at 8.2 ml disappeared upon addition of Nef inhibitor.
(TIF)

Figure S5 **Expression and localization of NI3-1 to NI3-12 in human cells.** (A) Localization of Nef and NIs in HeLa cells. GFP or Nef.GFP was co-expressed with an empty control vector or the indicated NIs and subjected to confocal microscopy analysis following fixation and anti-HA immunostaining. Presented are confocal sections of the middle of representative cells. (B) Western blot analysis of lysates of the cells shown in (A) using an anti-HA antibody for detection of the indicated NI proteins.
(TIF)

Figure S6 **Structural details of the complex interface between Nef_SF2_ and NI1-2.** (A) The central residues Y_89_VSWSPDD of the mutated RT loop tightly interact with helices α4 and α5 of Nef. Particularly the indol ring of W92_NI1-2_ performs multiple interactions with hydrophobic residues I91, F94, W117 and I118 of Nef. (B) A polar cluster between S93 and D95 of NI1-2 and K86 and D90 of Nef sustains the complex binding. (C) Interactions of the newly identified N-terminal helix (60–69) of Nef with its core domain structure. Tryptophane 61 undergoes tight interactions with L104, R110, I113 and L114 of the Nef core domain structure, covering the distal hydrophobic crevice of the α4 and α5 helices.
(TIF)

Figure S7 **NMR titration series of Nef_SF2_ (45-210, C59S, C210S) with inhibitor construct NI3-13.** Shown are ^15^N/^1^H HSQC spectra of ^15^N labeled Nef in the initial, unperturbed state (blue resonance signals), followed by addition of NI3-13 at a molar ratio to Nef of 0.5 (orange lines) and 1.2 (red line).
(TIF)
