# Supplementary figures and images for: Molecular Design, Functional Characterization and Structural Basis of a Protein Inhibitor Against the HIV-1 Pathogenicity Factor Nef
Source: PLoS One. 2011 May 20;6(5):e20033. doi: 10.1371/journal.pone.0020033 (PMC3098852; doi:10.1371/journal.pone.0020033)

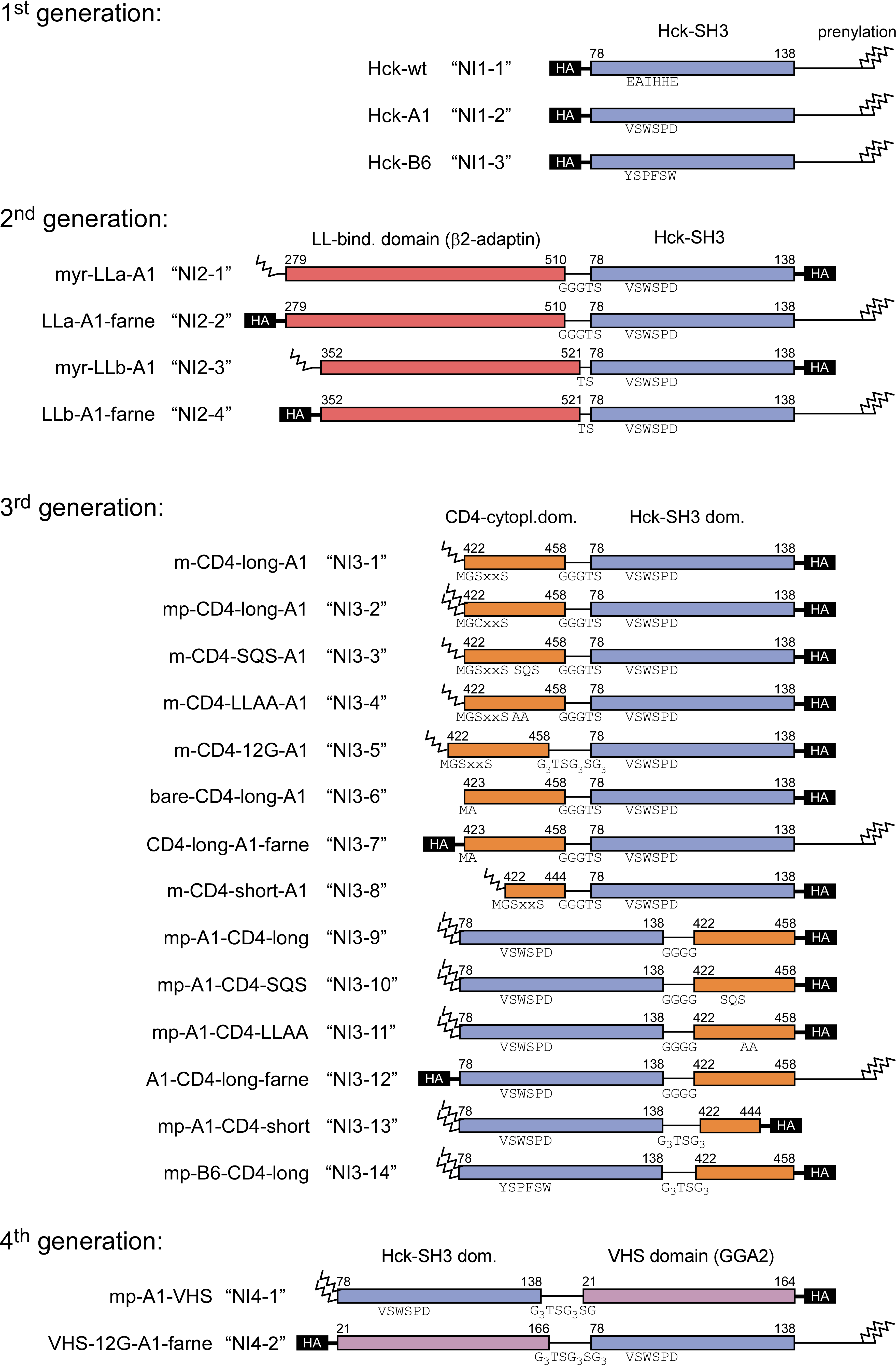

Supplement: Figure S1 — Schematic display of the four generations of inhibitor constructs used in cellular experiments. Domain boundaries, membrane targeting sites and sequence epitopes attached for antibody recognition in vivo are indicated. Domain boundaries for the human Hck SH3 domain are given according to the UniProt database entry P08631-1, described as isoform 1 of protein product p60-Hck. Residue numbering of human CD4 is assigned according to UniProt protein entry P01730. All protein products besides NI3-6 were targeted to cellular membranes either by an N-terminal myristoylation motif or a C-terminal farnesylation signal, partly in combination with an additional palmitoylation signal for increased membrane association, which is schematically indicated as carbon structure (zigzag lines). (TIF) [file pone.0020033.s001.tif]

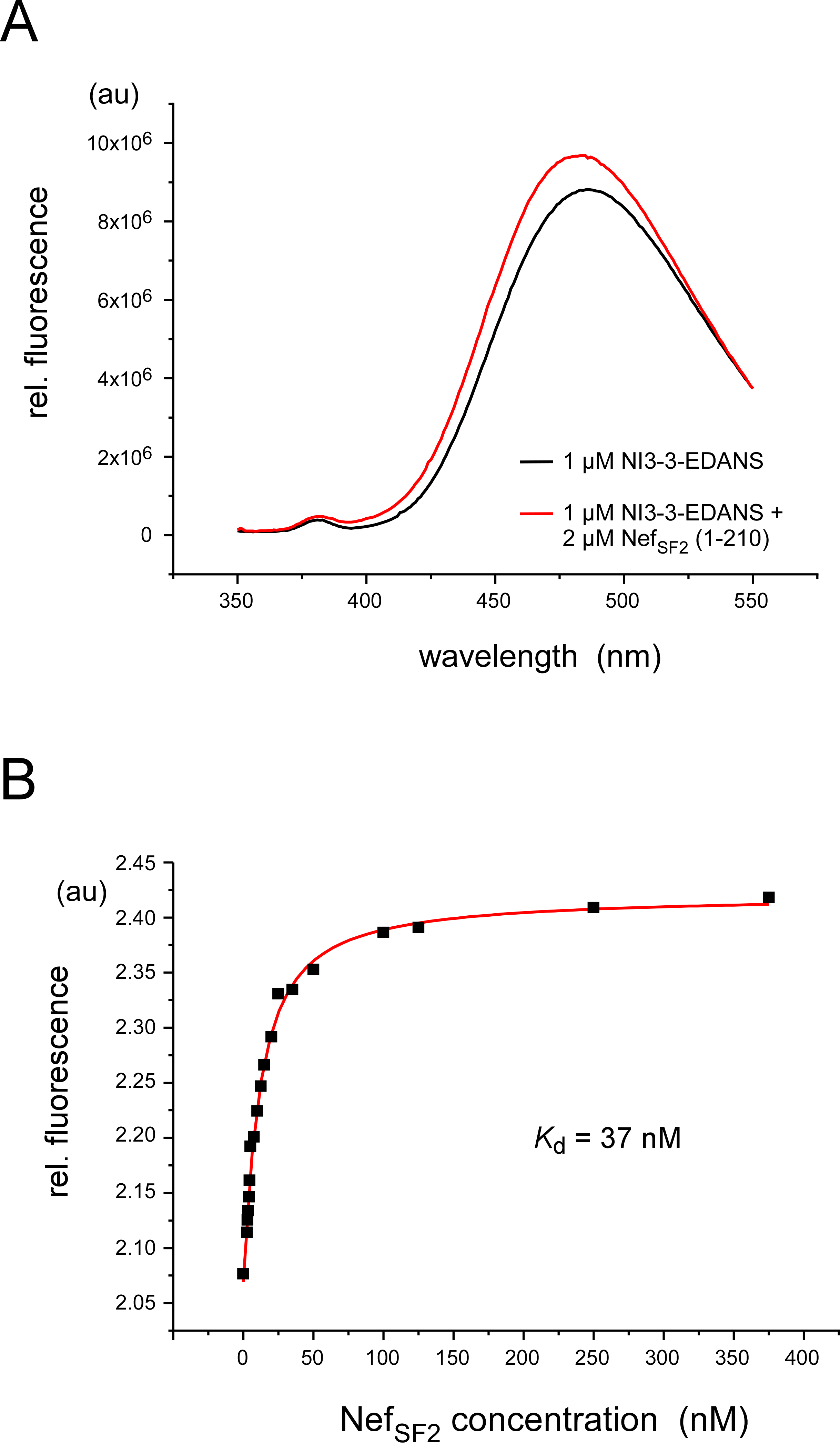

Supplement: Figure S2 — Fluorescence spectroscopy analysis of the binding interaction between Nef and NI3-3. (A) Emission spectra of fluorescence-labeled NI3-3 Hexim1 alone and in complex with Nef (1-210) indicate complex formation by an increase and shift in fluorescence emission. (B) Analysis of an equilibrium titration series of NI3-3-EDANS with increasing concentrations of Nef (1-210) indicated a dissociation constant K d of 37 nM. The relative fluorescence intensity was corrected for the dilution effects. (TIF) [file pone.0020033.s002.tif]

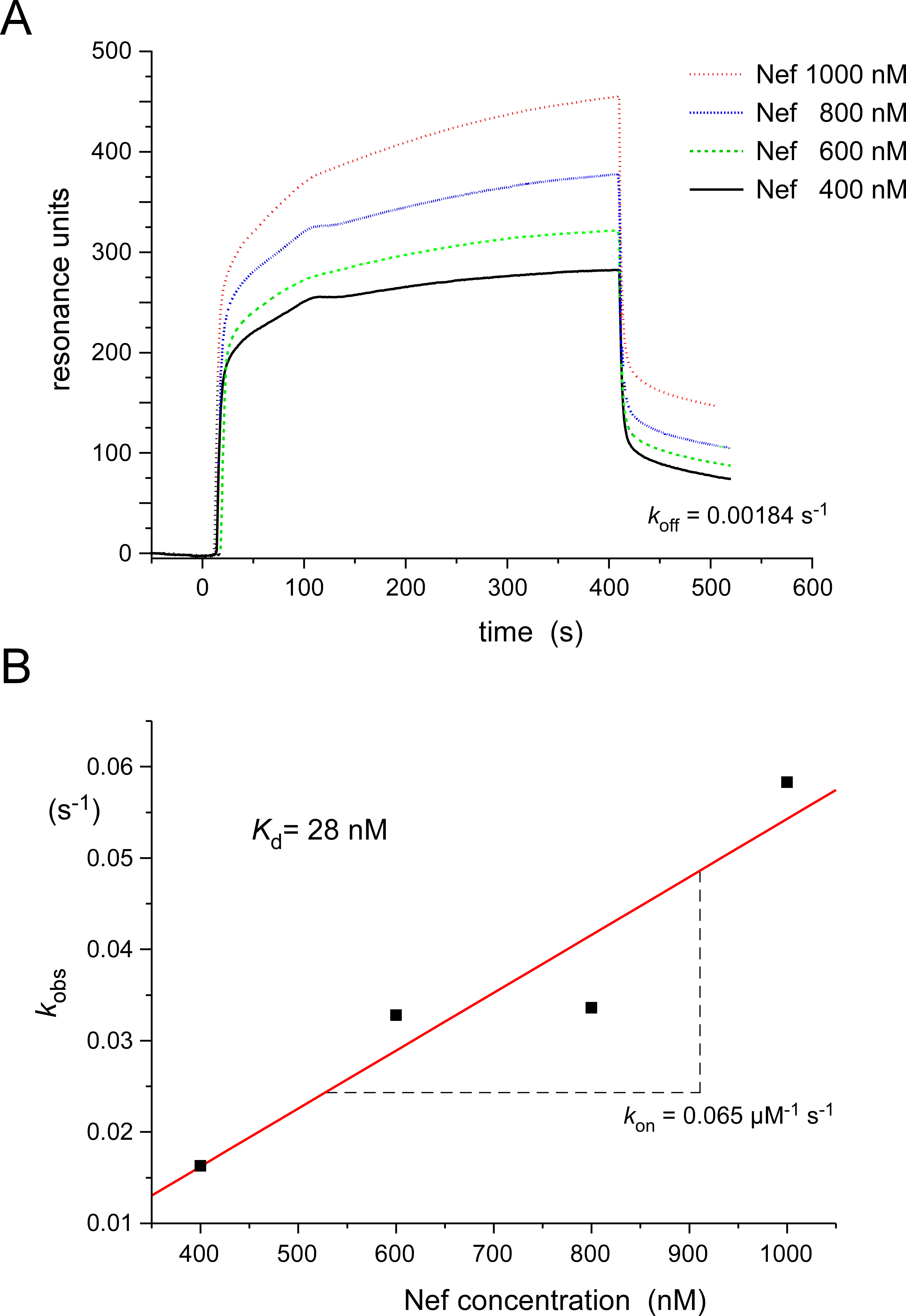

Supplement: Figure S3 — Surface plasmon resonance measurements of Nef binding to an inhibitor of the third generation. (A) NefSF2 (45-210) was floated at four different concentrations (0.4 to 1.0 µM) over GST-NI3-9 which was immobilized on the chip surface of the SPR biosensor. Following the association step Nef protein was washed off after 400 s by Guanidine hydrochlorid. (B) The concentration dependent analysis of the association and dissociation reaction revealed a dissociation constant of 28 nM for the Nef–GST-NI3-9 interaction. (TIF) [file pone.0020033.s003.tif]

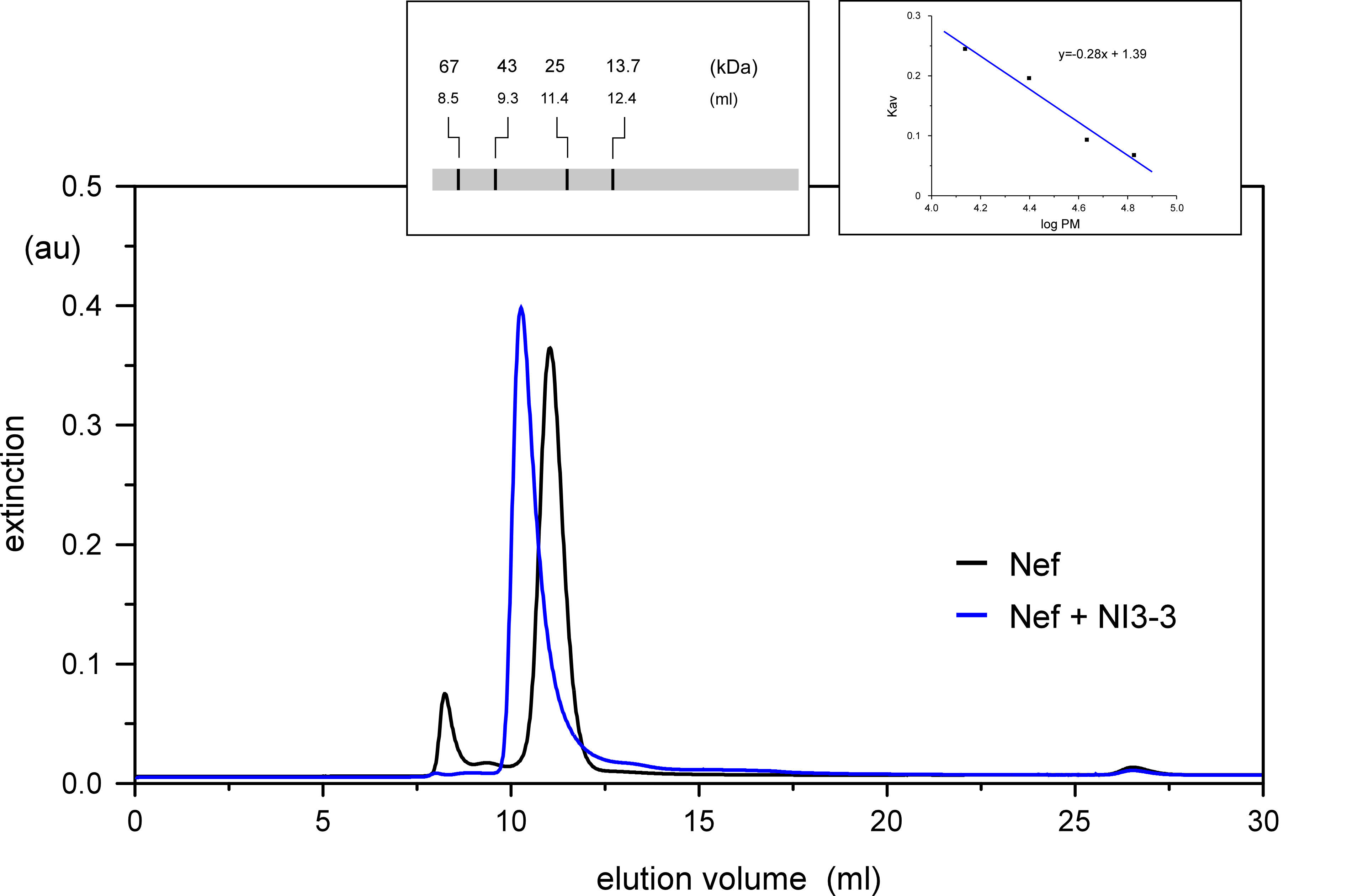

Supplement: Figure S4 — Size exclusion chromatography of Nef and the Nef–NI3-3 protein complex. While Nef (45-210) elutes at its expected size of ∼28 kDa as similarly observed before [53], the Nef–NI3-3 complex elutes at a molecular weight of approximately 38 kDa. These observations suggest a heterodimeric but not a heterotetrameric complex assembly, indicating that the gain in affinity by the CD4 fraction in NI3-3 is achieved by the interaction with the same Nef molecule. Note that the small portion of oligomerized Nef in the void volume at 8.2 ml disappeared upon addition of Nef inhibitor. (TIF) [file pone.0020033.s004.tif]

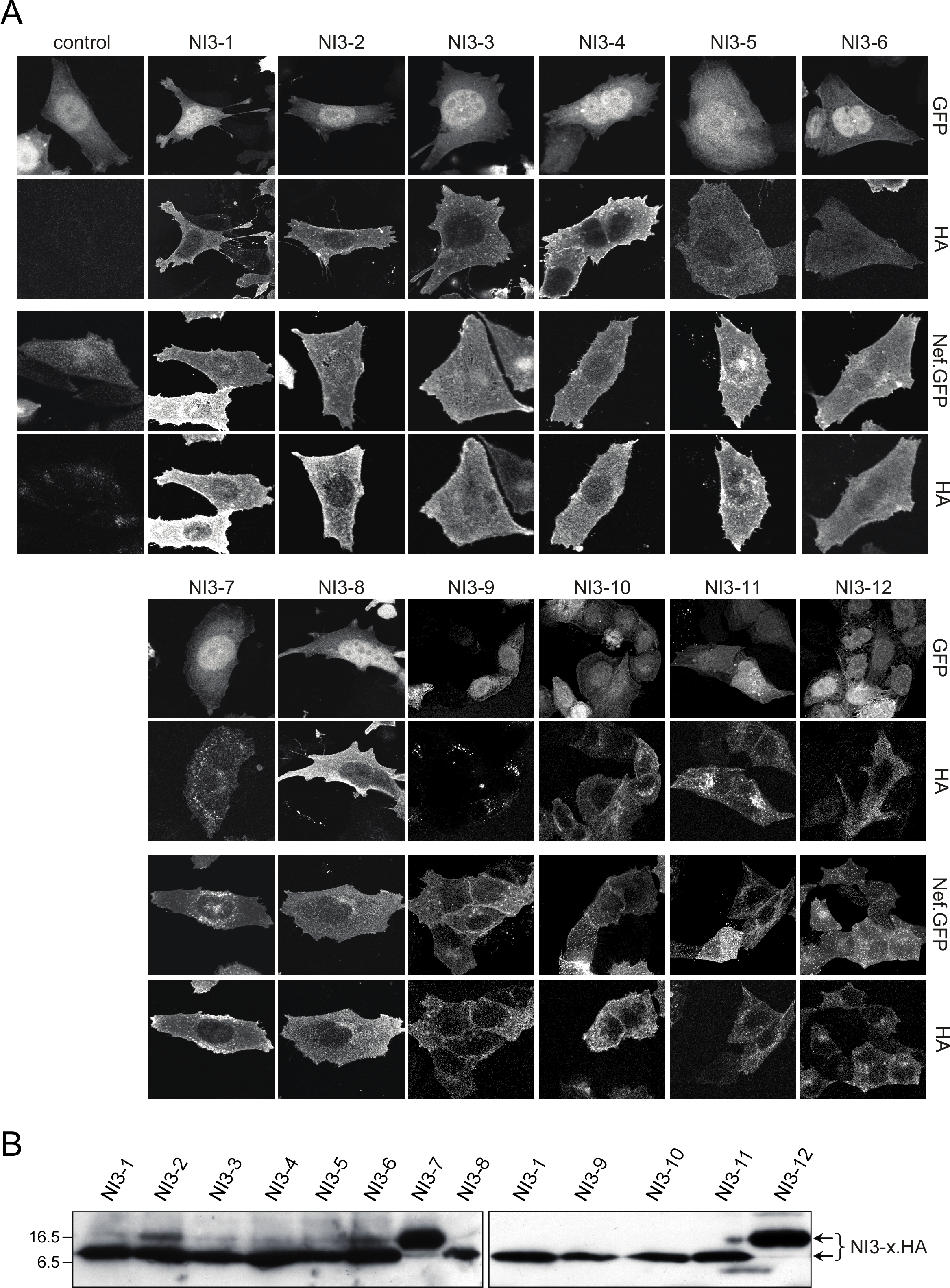

Supplement: Figure S5 — Expression and localization of NI3-1 to NI3-12 in human cells. (A) Localization of Nef and NIs in HeLa cells. GFP or Nef.GFP was co-expressed with an empty control vector or the indicated NIs and subjected to confocal microscopy analysis following fixation and anti-HA immunostaining. Presented are confocal sections of the middle of representative cells. (B) Western blot analysis of lysates of the cells shown in (A) using an anti-HA antibody for detection of the indicated NI proteins. (TIF) [file pone.0020033.s005.tif]

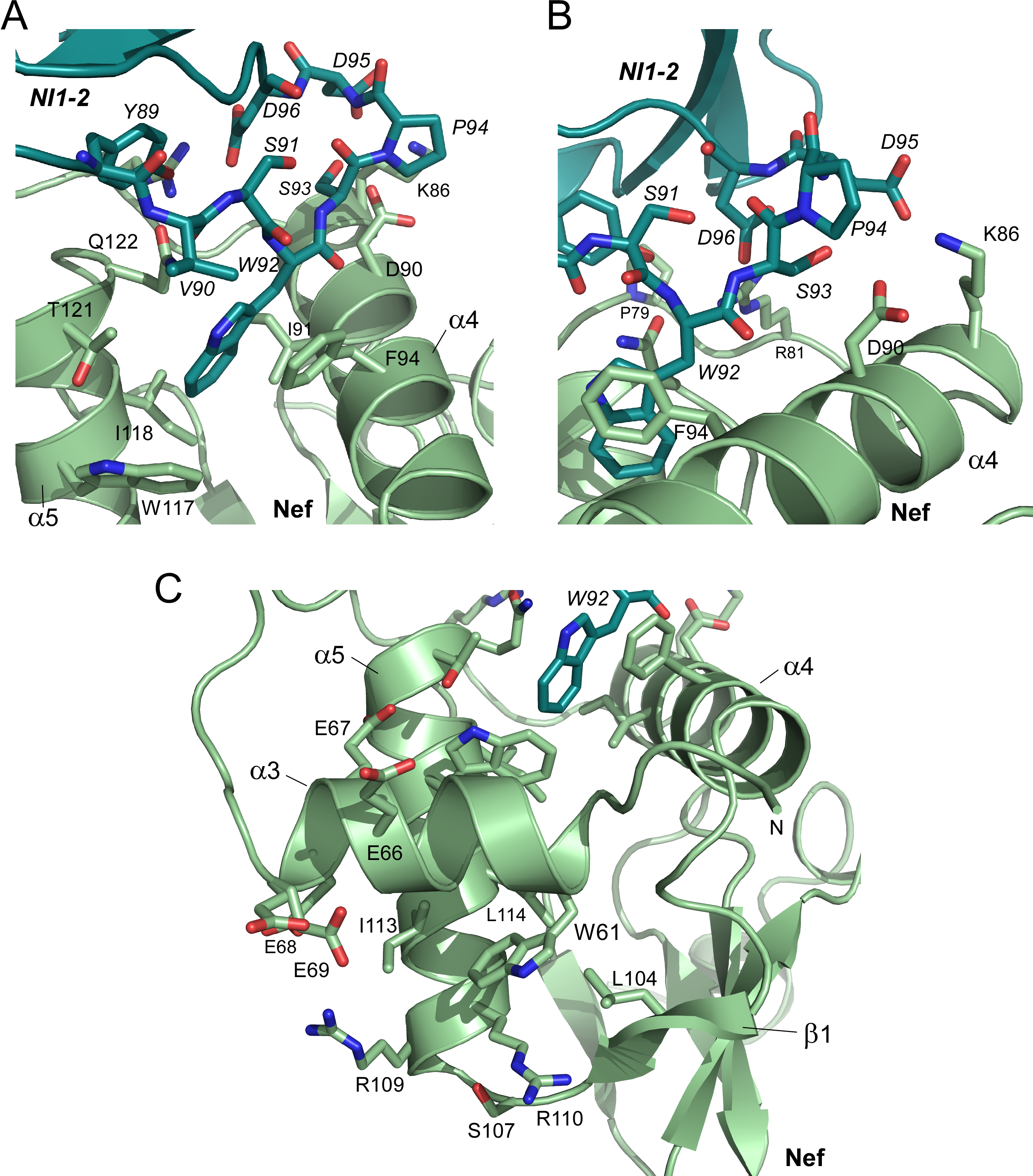

Supplement: Figure S6 — Structural details of the complex interface between NefSF2 and NI1-2. (A) The central residues Y89VSWSPDD of the mutated RT loop tightly interact with helices α4 and α5 of Nef. Particularly the indol ring of W92NI1-2 performs multiple interactions with hydrophobic residues I91, F94, W117 and I118 of Nef. (B) A polar cluster between S93 and D95 of NI1-2 and K86 and D90 of Nef sustains the complex binding. (C) Interactions of the newly identified N-terminal helix (60–69) of Nef with its core domain structure. Tryptophane 61 undergoes tight interactions with L104, R110, I113 and L114 of the Nef core domain structure, covering the distal hydrophobic crevice of the α4 and α5 helices. (TIF) [file pone.0020033.s006.tif]

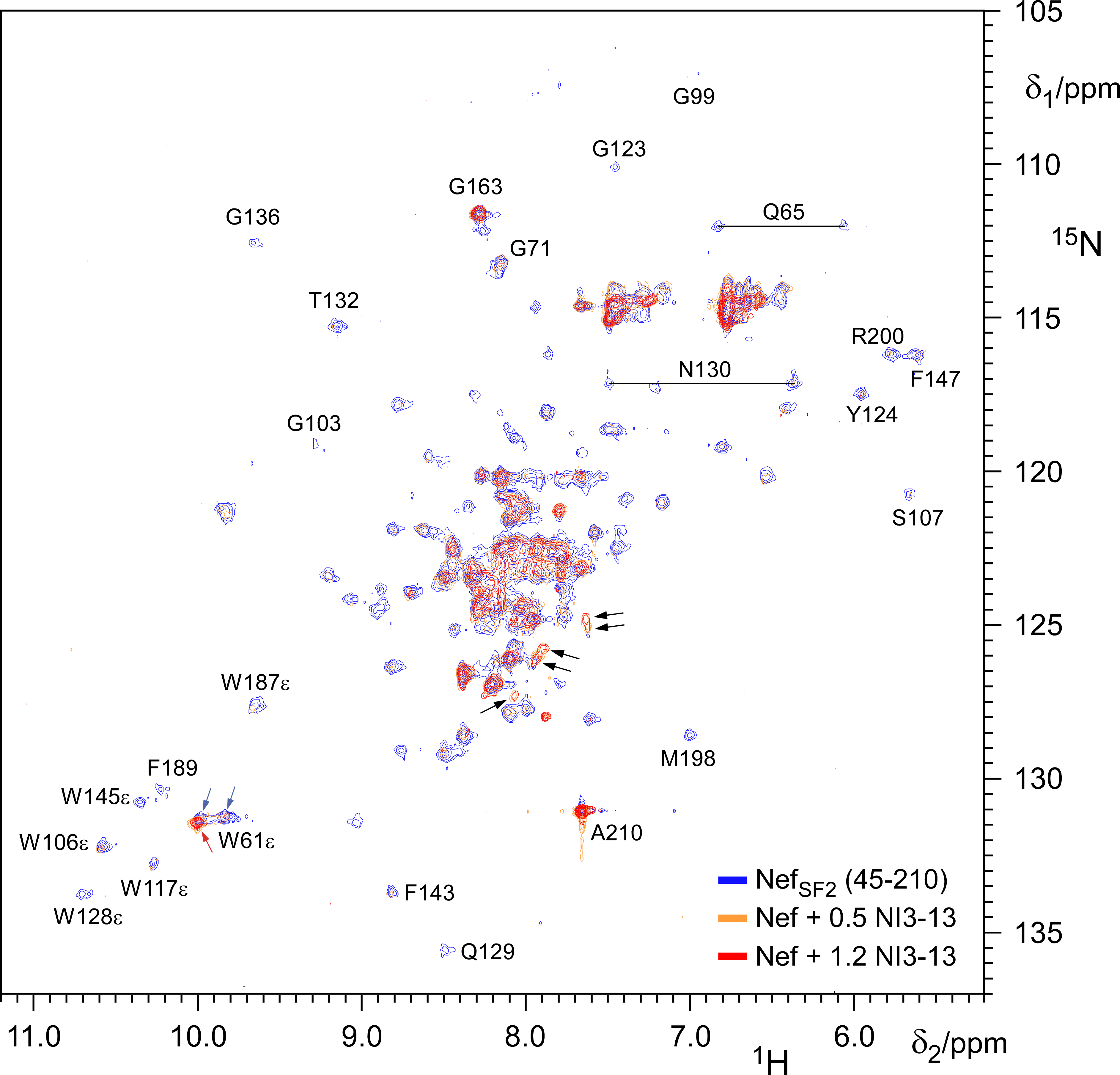

Supplement: Figure S7 — NMR titration series of NefSF2 (45-210, C59S, C210S) with inhibitor construct NI3-13. Shown are 15N/1H HSQC spectra of 15N labeled Nef in the initial, unperturbed state (blue resonance signals), followed by addition of NI3-13 at a molar ratio to Nef of 0.5 (orange lines) and 1.2 (red line). (TIF) [file pone.0020033.s007.tif]
